# Recovering the Genetic Identity of an Extinct-in-the-Wild Species: The Puzzling Case of the Alagoas Curassow

**DOI:** 10.1371/journal.pone.0169636

**Published:** 2017-01-05

**Authors:** Mariellen C. Costa, Paulo R. R. Oliveira, Paulo V. Davanço, Crisley de Camargo, Natasha M. Laganaro, Roberto A. Azeredo, James Simpson, Luis F. Silveira, Mercival R. Francisco

**Affiliations:** 1 Programa de Pós Graduação em Ecologia e Recursos Naturais, Universidade Federal de São Carlos, Rod. Washington Luís, CEP, São Carlos, SP, Brazil; 2 Programa de Pós Graduação em Diversidade Biológica e Conservação, Universidade Federal de São Carlos, campus de Sorocaba, Rod. João Leme dos Santos, CEP, Sorocaba, SP, Brazil; 3 Departamento de Ciências Ambientais, Universidade Federal de São Carlos, Campus de Sorocaba, Rod. João Leme dos Santos, CEP, Sorocaba, SP, Brazil; 4 CRAX—Sociedade de Pesquisa do Manejo e da Reprodução da Fauna Silvestre, rua Jarbas Camargo, Chácara Campestre, Contagem, MG, Brazil; 5 Seção de Aves, Museu de Zoologia da Universidade de São Paulo, CEP, São Paulo, SP, Brazil; Smithsonian Conservation Biology Institute, UNITED STATES

## Abstract

The conservation of many endangered taxa relies on hybrid identification, and when hybrids become morphologically indistinguishable from the parental species, the use of molecular markers can assign individual admixture levels. Here, we present the puzzling case of the extinct in the wild Alagoas Curassow (*Pauxi mitu*), whose captive population descends from only three individuals. Hybridization with the Razor-billed Curassow (*P*. *tuberosa*) began more than eight generations ago, and admixture uncertainty affects the whole population. We applied an analysis framework that combined morphological diagnostic traits, Bayesian clustering analyses using 14 microsatellite loci, and mtDNA haplotypes to assess the ancestry of all individuals that were alive from 2008 to 2012. Simulated data revealed that our microsatellites could accurately assign an individual a hybrid origin until the second backcross generation, which permitted us to identify a pure group among the older, but still reproductive animals. No wild species has ever survived such a severe bottleneck, followed by hybridization, and studying the recovery capability of the selected pure Alagoas Curassow group might provide valuable insights into biological conservation theory.

## Introduction

Natural hybridization and gene introgression play a significant role in the evolution of many taxa [1–3]. However, human activities such as habitat modification and the introduction of exogenous plant and animal species can artificially eliminate reproductive isolation between organisms, leading to the occurrence of anthropogenic hybridizations [4–7]. The resulting disruption of adaptive complexes has become a major concern for conservation practitioners because it has jeopardized a growing number of populations and species worldwide and is especially harmful when a rare species comes into contact with a more abundant one [4, 5, 8]. Although most attention has been devoted to the effects of human-mediated hybridizations on free-ranging species and subspecies, a number of works have revealed its importance for ex situ breeding programs of endangered taxa [9–12], and here, we also address such cases as components of anthropogenic hybridization.

The occurrence of anthropogenic hybridization in captivity can be either intentional, when i) populations that have been through severe bottlenecks can benefit from gene introgression to tackle inbreeding depression [13], ii) fertile individuals of only one sex remain, both in the wild and in captivity [14], or iii) the sexual rate is unbalanced, and individuals of the exceeding sex are crossed with a related species to maintain sexual libido while individuals of its own species are not available (present study), or unintentional, when i) founders are obtained or confiscated from animal keepers that have permitted or stimulated hybridizations [10], ii) cryptic species are kept in the same breeding facilities [9, 11, 15], iii) taxonomically unresolved taxa are combined [16, 17], or iv) hybrids were already present in the wild populations that founded the captive program [16].

Unintentional anthropogenic hybridization becomes especially harmful when the hybrids are fertile, which implies different levels of introgression to complete admixture [5]. In these cases, after a number of backcrosses, hybrids become morphologically indistinguishable from the parental species, making the conservation of a threatened taxa more difficult, and the use of molecular genetic markers is often the only way to assign admixture levels [9, 11, 12]. Despite the theoretically controlled captive conditions, recent genetic analyses have proven that unintentional gene introgressions have affected even some of the most traditional ex situ conservation programs, including the American Bison [10], Chimpanzees [11], and Tigers [9], suggesting that other programs might also be vulnerable to this problem, especially ones that are established in countries in which captive management is not regulated by official organizations.

The Alagoas Curassow (*Pauxi mitu*, Cracidae), is an endemic bird from the Atlantic Forest of northeastern Brazil and has become a symbol for the conservation of this biome because it is one of the most threatened birds in the world. It is believed that this species vanished from the wild in the early 1980s, due to deforestation and overhunting [18, 19], although the last confirmed record in nature was in 1979, when five individuals were captured in Alagoas State and brought into a private breeding facility located in Rio de Janeiro city. Three of these individuals have reproduced (one male and two females) and founded the current captive population [19], constituting one of the most severe bottlenecks ever documented for a wild animal that has survived, only comparable to the Mauritius Kestrel [20, 21]. In 1990, when the population reached 19 individuals (12 males and seven females), the excess males were hybridized with females of the closely related Razor-billed Curassow (*P*. *tuberosa*), a more common species from the Amazon forests. Hybrids of both sexes were fertile (see [22, 23]) and proved to be able to reproduce either among themselves or with parental Alagoas Curassows, whereas backcrosses with Razor-billed Curassows were never permitted. Gene introgression was attempted first to form a ‘‘back up” population with a certain amount of admixture that could be used in the future to address potential inbreeding depression problems [24] and second to keep the excess males sexually active, as it is commonly reported by managers that curassows born in captivity lose their libido if they are not paired at the beginning of their fertile life. Although hybridization was intentional, in subsequent years, pure individuals, F1 hybrids, later generations of hybrids, and backcrosses were deliberately produced without pedigree recordings. In 1999, there were 44 individuals, and all of them were transferred into two new breeding facilities from Minas Gerais State. Since then, the use of Razor-billed Curassows has ceased, and crossings have prioritized individuals with the Alagoas phenotype, although specimens with hybrid phenotypes have often been crossed with typical Alagoas Curassows when one member of a pair was infertile.

In a preliminary study, [25] demonstrated that a number of individuals with introgressed mtDNA could not be distinguished from the parental species based on their morphology. In 2008, when the total population reached more than 100 individuals, Brazilian governmental authorities created the ‘‘National Action Plan for the Conservation of the Alagoas Curassow”, and the uncertainty about admixture levels has opened an important debate regarding whether this group of animals has conservation value, thus putting at risk the ex situ breeding program and its financial support, which mostly comes from the owners of the aviaries and from non-governmental organizations. Thus, identifying whether pure individuals, or individuals with acceptable levels of admixture, were still present in the population became a major challenge for the conservation of the Alagoas Curassow. In the best-case scenario, the genetic identity of this species would be recovered, and the breeding program could be reorganized to preserve its genome integrity.

As only female Razor-billed Curassows have been used in crossings with Alagoas Curassows, molecular markers with matrilineal inheritance patterns, such as mtDNA diagnostic sites, would be efficient to assign a hybrid origin to F1 and later-generation descendants from those hybrid females, as they would all present a Razor-billed mtDNA molecule (see also [25]). In contrast, mtDNA could not detect hybridization in backcrossed animals descending from female Alagoas Curassows, thus indicating the need for biparental nuclear markers to assess admixture levels. However, three main aspects make this case particularly puzzling: i) nuclear markers can often assign an individual a hybrid origin only within a few backcross generations [5, 26, 27], and here at least eight generations have passed since the beginning of the hybridizations; ii) in contrast to most hybridization analyses, in which sets of putatively pure individuals of each parental species are available to parameterize the admixture levels of a number of organisms with unknown ancestry [9, 27–30], here the existence of pure Alagoas Curassows was totally uncertain, and approximately half of the population presented Razor-billed phenotypic traits, suggesting that the lack of parameterization could reduce the analysis performance [31]; and iii) analyses based on biparental molecular markers become more efficient and accurate when diagnostic alleles or loci are present [5, 32]. However, no tissue samples were kept from the three founder Alagoas Curassows, which died during the 1990s, which has impeded the identification of diagnostic alleles and loci.

Then, we applied an analysis framework that combined morphological diagnostic traits, Bayesian clustering analyses using 14 unlinked microsatellite loci, and mtDNA control region haplotypes in an attempt to assess the ancestry of each animal. To minimize the effects of the potential lack of parameterization, Bayesian procedures were performed using a sample of Razor-billed Curassows as prior information [33–35], and a pre-defined Alagoas group was created by selecting individuals that presented very low probabilities of Razor-billed introgression. Then, these individuals were used to parameterize the subsequent analyses (see also [36, 37]). The risks of errors associated with our estimations were assessed *a posteriori* using simulated classes of hybrids created from the Razor-billed sample and from the pre-defined Alagoas group. The presence of loci and alleles that could be diagnostic of each species, as well as genetic distance, were also assessed *a posteriori*. Although this study focused on the Alagoas Curassow, our analysis framework has a clear relevance to the conservation of other taxa that might face a similar situation of complete genetic identity uncertainty. Our specific aims are (1) to determine whether the potentially pure Alagoas Curassow still exists, (2) to classify individuals with mixed ancestry into categories, and (3) to provide general recommendations for species management.

## Materials and Methods

### Bird sampling and DNA extraction

From 2008 to 2012, we collected blood samples from all of the living birds presenting phenotypic characteristics of the Alagoas Curassow (n = 85), and from the birds presenting hybrid morphology (n = 63). These animals were distributed in two Brazilian aviaries: CRAX—Sociedade de Pesquisa do Manejo e da Reprodução da Fauna Silvestre, and Criadouro Científico e Cultural de Poços de Caldas. A total of 33 blood samples from Razor-billed Curassows were also collected, 30 from aviaries and Brazilian zoos and 3 from wild-caught animals deposited in the scientific collection of the Museu de Zoologia da Universidade de São Paulo (MZUSP). All captive animals were banded using metal rings for permanent identification. Blood was taken by venipuncture from the brachial wing vein and stored in 100% ethanol at -20°C. Total genomic DNA was extracted using a conventional phenol/chloroform/isoamyl alcohol 3:3:1 protocol, and samples were stored in EDTA buffer at 50 ng/μl concentration. Blood sampling and collection methods were authorized by the responsible Brazilian Federal Government Institution (Ministério do Meio Ambiente, Instituto Chico Mendes de Conservação da Biodiversidade, SISBIO/ICMBio: permits#15960–1 and 16027–1). The aviary owners permitted access to the animals, and the collect of all samples were also approved by the Ethics Commission on Animal Use of the Instituto de Biociências da Universidade de São Paulo (CEUA–IBUSP).

### Morphological identification

**Fig 1:**
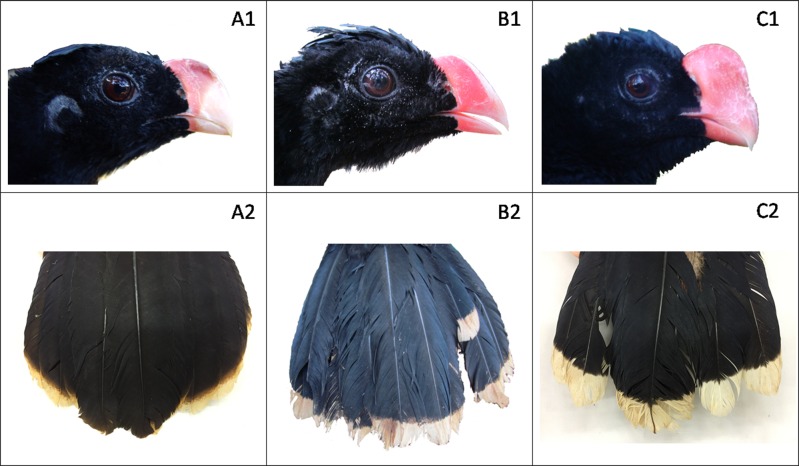
Diagnostic morphological characteristics of Alagoas Curassow. Pure Alagoas Curassow (A1 and A2), hybrid (B1 and B2), and Razor-billed Curassow (C1 and C2).

Alagoas and Razor-billed Curassows are clearly distinguishable by four diagnostic morphological traits: 1) color of the bill, which is red at the base becoming whitish towards the tip in the Alagoas and uniformly red in the Razor-billed Curassow; 2) auricular patch, which is bare in the Alagoas and totally feathered in the Razor-billed Curassow; 3) tail tip color, which is pale tawny in the Alagoas and pure white in the Razor-billed Curassow, and 4) the central rectrices pair, which is all-black in the Alagoas and presents white tips in the Razor-billed Curassow [19]. All of the animals had their heads and tails photographed, and we qualitatively assigned individuals as hybrids when they presented one or more of the following characteristics: uniformly red bill, partially or totally feathered ear, white instead of pale tawny tail tips, and central rectrices with some whitish color in the tip (Fig 1).

### Microsatellite genotyping

Individual multilocus genotypes were obtained using a set of 14 unlinked microsatellite loci, nine being species-specific (*Pauxi* 1–4, *Pauxi* 1–13, *Pauxi* 1–30, *Pauxi* 1–37, *Pauxi* 2–2, *Pauxi* 2–7, *Pauxi* 2–30, *Pauxi* 3–1, and *Pauxi* 3–4), and five loci isolated for the Jacutinga (*Aburria jacutinga*, Cracidae) (*Aburria* 21, *Aburria* 22, *Aburria* 36, *Aburria* 48, and *Aburria* 49). Details on loci isolation and characterization, as well as PCR conditions, are described in [38] and [39]. Amplifications were performed using fluorescently labeled primers, and products were run on an automated sequencer (ABI 3730). Allele sizes were scored using the software GeneMarker2.4.0 (Softgenetics). Raw genotypic data are presented in S1–S3 Files.

### Microsatellite Bayesian clustering analyses

#### First stringent analyses

To estimate the extent of admixture of each individual, first we used the Bayesian MCMC clustering method implemented in the software Structure 2.3.4. [34, 35]. In this procedure, the unknown number of genetic clusters (*K*), and the likelihood (*q* value) that each individual could be assigned to one of the populations (or species) is estimated simultaneously in such a way as to achieve HW and linkage equilibrium. To find the most appropriate *K*, we ran 10 replicates of each *K* (from *K* = 1 to *K* = 5) using admixture model with correlated allele frequencies, and default parameter settings. We adopted 1,000,000 MCMC iterations (with 100,000 discarded as burn-in), and the best *K* was defined using the method of [40], implemented in the software Structure Harvester [41], which confirmed *K* = 2 as expected for our sample. Because we were not sure if pure Alagoas Curassows were still present in the population, after defining *K* we ran a stringent analysis in an attempt to compose a pre-defined Alagoas Curassow sample that could be used to parameterize the subsequent analyses (see also [36, 37]). To improve the accuracy of this inference, Razor-billed Curassows were used as prior population information, with default parameter settings. This ancestry model is useful when some individuals of known ancestry (here the Razor-billed Curassows) are used to classify individuals of unknown origin [9, 34, 36]. Because a large proportion of hybrids in relation to parental individuals tend to weaken the estimation [31], animals identified as hybrids based on morphology were not considered in the stringent body of analyses. We adopted 1,000,000 MCMC iterations and a burn-in period of 100,000 reps. This analysis was ran in 20 replicates and *q* values were aligned with Clumpp, using the *FullSearch* algorithm, which is the most probable to find the optimal *q* values obtained after multiple runs of Structure [42]. Individuals were considered to have mixed ancestry when they had less than 99% (*q* < 0.99; threshold value) assignment probabilities to belong to the Alagoas Curassow [37].

We also used NewHybrids, a Bayesian model-based MCMC framework that estimates the posterior probability (*Q*) of each individual to belong to user-defined genotypic classes [33]. In this step, we used default genotypic classes (parental species A, parental species B, F1 hybrid, F2 hybrid, and F1 backcrosses). Razor-billed Curassows were marked as individuals of known genotypic category (z0) to improve the power of the inference, and to obtain the most stringent result different combinations of Jeffreys and Uniform priors were tested [33]. We ran 1,000,000 MCMC iterations (discarding 100,000 as burn-in), and three replicates were performed to check for potential variations in *Q* values. Again, individuals were considered to have mixed ancestry when they had less than 99% posterior probabilities of belonging to Alagoas Curassow.

#### Assessing the power of models and markers

To assess the power of the models and markers, and to define the best threshold value for Structure analyses, we used Hybridlab 1.0 [43] to generate expected genotypes for the different parental and hybrid classes [29, 44]. This software creates multilocus genotypes through randomly selecting one allele from each of two pre-defined parental populations based on their allelic frequencies. The 33 Razor-billed Curassows and the individuals assigned as pure Alagoas Curassows in the most restrictive of the stringent analyses, and in mtDNA analysis (the pre-defined Alagoas Curassows) were used to generate 100 samples of each parental and hybrid class: Alagoas Curassow, Razor-billed Curassow, F1, F2, F3, and first, second, third, and fourth generations of backcrosses with Alagoas Curassow. Backcross with Razor-billed Curassow was not considered, as this category of hybridization did not occur.

Using Structure, the ancestry levels of the simulated individuals were estimated using a Prior Population Information model. Razor-billed Curassows and the pre-defined Alagoas Curassows were assigned POPFLAG = 1, indicating that they were used to estimate allele frequencies, and simulated samples were assigned POPFLAG = 0, indicating unknown ancestry. A possible threshold value effect was evaluated by arbitrarily using 95% (*q* < 0.95), 98% (*q* < 0.98), and 99% (*q* < 0.99).

In NewHybrids, Razor-billed Curassows and the pre-defined Alagoas Curassows were also marked as individuals of known genotypic category (z0 and z1, respectively). Different combinations of Jeffreys and Uniform priors were compared, and simulated individuals were assigned to a class when they had ≥ 70% probability of belonging to a single genotypic class [45]. Then, to assess the power of the markers and models we evaluated the percentage of individuals of each simulated class that were correctly assigned as pure and hybrids by each software, independently of attributing them to specific hybrid classes. Numbers of iterations and replicate approaches were the same used above.

#### Attribution of morphological hybrids to categories

Finally, we assessed the ancestry level of those individuals identified as hybrids based on morphology. Using Structure, we adopted Prior Population Information model, and both the pre-defined Alagoas Curassows and the Razor-billed Curassows were assigned POPFLAG = 1, while the others were assigned POPFLAG = 0. Then, resulted *q* values obtained with Clumpp after 20 replicates were compared to the ranges of *q* values obtained for the different simulated hybrid classes in an attempt to assign them to specific hybrid classes. With NewHybrids, Razor-billed Curassows and the pre-defined Alagoas Curassows were again marked as individuals of known genotypic category (z0 and z1, respectively), and we adopted the most efficient prior combination observed in the analyses with the simulated dataset. Numbers of iterations were the same described above.

### mtDNA analyses

Maternal ancestry was assessed by the presence of mtDNA haplotypes of the Razor-billed Curassow in individuals assigned to the Alagoas Curassow in morphology and in Bayesian clustering analyses [25, 46]. To determine the mtDNA type of each individual, we PCR-amplified the third domain and part of the central domain of the control region using the primers L774-GAGACGGTTTGCGTATATGC [47], modified based on the sequence AY145306.1 of Bare-faced Curassow, *Crax fasciolata*, from GenBank) and H1251-TCTTGGCATCTTCAGTGCCATGC [48]. Numbers represent the position in the *Gallus gallus* map [48].

PCR reactions were performed using an Eppendorf MasterCycler Gradient thermal cycler, in a 25 μl volume containing 100 ng of DNA, 150 μM of each dNTP, 6,5 μl of amplification buffer (200 mM Tris-HCl, pH 8.4, and 500 mM KCl; Promega), 0.25 μM of each primer, 2 mM of MgCl_2_, 2.5 μl of BSA 25μg/ml, and 1 U *Taq*-Polymerase (Promega). Cycling specifications were 94°C (5 min), 30 cycles of 94°C (30 s), 50°C (30 s), and 72°C (40 s), followed by a final extension of 72°C (10 min). Before sequencing, the PCR products were purified using the *Wizard SV Gel and PCR Clean-Up System*, Promega. The light strains were resolved on an ABI 3730 automated sequencer using the BigDye® Terminator v3.1 Cycle Sequencing Kit. Sequences were aligned automatically using the CLUSTAL W procedure [49] implemented in Mega version 6 [50].

We amplified 11 samples of Razor-billed Curassows (including the three animals captured in the wild) and the individuals assigned to Alagoas Curassows in two different steps: first, we amplified samples of individuals assigned to Alagoas Curassow in the stringent analyses by Structure, and by NewHybrids most restrictive prior combination, and second, by Structure using 98% threshold, which proved to be the best approach to identify hybridization using our markers (see results bellow). Thus, we manually diagnosed for species-specific haplotypes and polymorphic sites that could indicate ancient maternal ancestry of the Razor-billed Curassow in individuals assigned to the Alagoas Curassow, and we produced a Median Joining network [51] using the software PopART (http://popart.otago.ac.nz).

### Analysis of genetic differentiation

Genetic differentiation between the two species, numbers of alleles, and the presence of diagnostic loci or alleles were assessed *a posteriori* by comparing the 33 Razor-billed Curassows with the group of individuals assigned to the Alagoas Curassow based on microsatellite and mtDNA analyses. The level of genetic differentiation was estimated by the Fixation Index (*F*_ST_) [52], and its significance was assessed by testing whether the genotypic distribution was identical between species using the log-likelihood (G) based Exact Test, implemented in FSTAT v. 2.9.3.2 [53], after 10,000 permutations. To visualize the genetic relationships among parental and admixted individuals based on multilocus microsatellite genotypes we used a Factorial Correspondence Analysis (FCA), as implemented in the software Genetix, version 4.05.2 [54]. This is a multivariate approach that produces new variables and plot each individual in a two- or tri-dimensional space, without any a priori information (see [55, 56]).

### Genetic identification of sex

As curassows of the genus *Pauxi* do not present sexual dimorphism, all of the individuals were sexed by amplification of the homologous copies of the CHD (chromo-helicase-DNA-binding) gene, located in the Z and W sex chromosomes, using the primers P2/P8 [57], and P0 [58] in the same PCR reactions. PCR reactions were performed according to [59], and the amplification products were run in 3% agarose gels.

## Results

### Morphological and bayesian analyses

**Fig 2:**
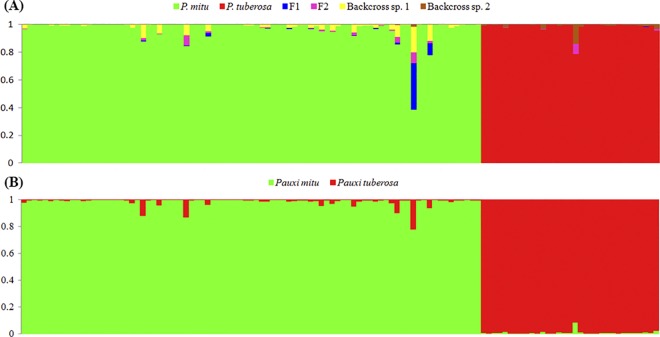
Proportional membership of potential Alagoas Curassows (*Pauxi mitu*), and Razor-billed Curassows (*P*. *tuberosa*) obtained with 14 microsatellite loci. **(A)** First stringent analysis performed in NewHybrids. **(B)** First stringent analysis performed in Structure.

We sampled all of the hybrids and potential Alagoas Curassows that were alive between 2008 and 2012, totaling 148 animals. Of these animals, 85 (57.4%) could be morphologically assigned to the Alagoas Curassow. The stringent preliminary analysis performed in NewHybrids using Uniform X Jeffreys prior combination was the most restrictive, revealing that 60 of the 85 individuals presenting the Alagoas Curassow phenotype had assignment probabilities of 99% or more of belonging to the Alagoas genotypic class. The analysis performed in Structure was the second most stringent, with 63 individuals assigned to Alagoas cluster with 99% threshold (Fig 2). Individual assignment probabilities obtained with both softwares and prior combinations are summarized in S1 Table. Three of the 60 individuals indicated as pure by NewHybrids, and six of the 63 individuals indicated as pure by Structure presented Razor-billed Curassow mtDNA (see results below), in such a way that after mtDNA elimination, both softwares indicated the same 57 animals to composed the pre-defined Alagoas Curassow group used in the subsequent analyses.

The analyses performed with the simulated dataset revealed that Newhybrids was more efficient than Structure to assign pure Alagoas and Razor-billed Curassows to their respective classes, as Structure could not attributed a pure origin to a few simulated parental individuals (Table 1, S2 Table). Both softwares were efficient to detect F1 hybrids, but NewHybrids often could not attribute F2 and F3 individuals to a single class with 70% or more posterior probability, independently of prior combinations. Further, in NewHybrids 7% of first, 68% of second, 97% of third, and 100% fourth backcross generations were erroneously attributed to Alagoas Curassow with the best prior combination according to the simulations (Jeffreys x Jeffreys) (Table 1, S2 Table). Then, Structure approach was superior to assign a hybrid origin to admixted individuals (Table 1), and we adopted this software to draw our conclusions, despite the small risk of assigning an admixted origin to a pure individual. In Structure, a threshold value effect was detected, as using the 95% level we had 100% efficiency of F1, F2, and F3 hybrids detection, but it was not safe to identify backcrossed individuals as hybrids. Using the 98 or 99% levels, 100% of the individuals could be assigned as hybrids up to the second backcross generation, dropping sharply in the third generation with a limit of 98% (Table 1). Thus, we concluded that the 98% cut limit was more efficient than the 99% cut for detecting late backcross generations in the simulated dataset because it might reduce the probability of assigning a pure Alagoas Curassow as a hybrid. As a result, nine individuals eliminated by Structure in the first stringent analysis, not presenting Razor-billed mtDNA (see bellow), were reincorporated in the Alagoas group, totaling 66 individuals.

**Table 1:** Bayesian analyses with 100 simulated genotypes of each parental and hybrid class, indicating the percentages of simulated individuals correctly assigned as parental or hybrid, independently of hybrid class attribution for both Structure and NewHybrids. In Structure analyses, potential threshold effect was evaluated using 95, 98 and 99% threshold levels, using K = 2. In NewHybrids, modeling effect was assessed by testing different combinations of Jeffreys (Jef), and Uniform (Un) priors.

Simulated category	Structure			NewHybrids			
	95% level	98% level	99% level	Jef x Jef	Jef x Uni	Un x Un	Un X jef
*Pauxi mitu*	98%	97%	96%	100%	100%	100%	100%
*Pauxi tuberosa*	99%	94%	88%	100%	100%	100%	100%
F1	100%	100%	100%	100%	100%	99%	99%
F2	100%	100%	100%	0	0	0	0
F3	100%	100%	100%	0	0	0	0
Backcross 1	99%	100%	100%	84%	66%	42%	78%
Backcross 2	82%	100%	100%	8%	0	0	0
Backcross 3	49%	67%	96%	0	0	0	0
Backcross 4	31%	42%	65%	0	0	0	0

The Structure analysis using both Alagoas group and the Razor-billed Curassows as prior population information to assess the ancestry level of the 63 individuals identified as hybrids based on morphology revealed that none of them were assigned to the Alagoas Curassow, and their probability values obtained with CLUMPP after 20 replicates varied from 0.027 to 0.849 (0.320 ± 0.292) (S3 Table). Due to the highly overlapping ranges in expected *q* values found for the different simulated hybrid classes (S2 Table), most of the hybrids could be assigned to at least two hybrid categories, such that we could not classify them into hybrid classes. Using NewHybrids with Razor-billed Curassows and the pre-defined Alagoas Curassows as individuals of known genotypic category (z0 and z1, respectively), and Jeffreys x Jeffreys prior combination (the best obtained with the simulated data), all of the morphological hybrids were erroneously attributed to Alagoas Curassow (S3 Table).

### mtDNA diagnosis

**Fig 3:**
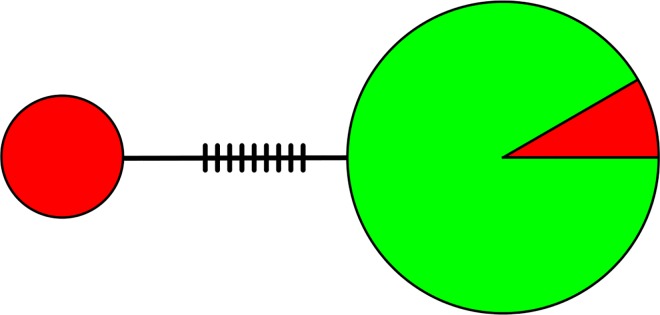
Median Joining network based on 311 bp of the mtDNA control region for 11 Razor-billed Curassows (left circle), and for 72 animals assigned to Alagoas Curassow based on the stringent analyses and Structure analyses using 98% threshold (right circle), with circle sizes reflecting the numbers of individuals. Green and red colors represent Alagoas and Razor-billed Curassow haplotypes, respectively, indicating Razor-billed maternal ancestry for six individuals assigned to Alagoas Curassow based on morphology and microsatellites. Hatches represent unobserved haplotypes.

The mtDNA PCR-amplification resulted in a 311 bp product. Our alignment showed two distinct haplotypes with nine polymorphic sites, all of which were parsimoniously informative nucleotide substitutions (Table 2). The amplification products were certainly of mitochondrial origin because they generated single PCR products per individual, and the sequences were unambiguous and similar to homologous products deposited in GenBank. Furthermore, a detailed study on the cracid mitochondrial genome failed to find nuclear copies of the control region [47]. We are confident that the haplotypes are species-specific because one was fixed in the Razor-billed Curassow group, and the other was predominant in the group of animals assigned to Alagoas based on the stringent analyses, and on Structure using 98% threshold. Of the 72 individuals of the Alagoas group, six presented the Razor-billed Curassows haplotype, indicating old maternal Razor-billed Curassow ancestry (Fig 3). One sequence of each haplotype was deposited in GenBank under accession numbers KU170649 and KU170650.

**Table 2:** Numbers of Razor-billed Curassows (*Pauxi tuberosa*), and of animals assigned to the Alagoas Curassow (*P*. *mitu*) based on morphology and microsatellite analyses, presenting each of two mtDNA control region haplotypes. Vertical numbers correspond to positions in the sequences deposited in GenBank (accession numbers KU170649 and KU170650) containing diagnostic variations.

Haplotypes	Numbers of individuals		Diagnostic site positions								
	*P*. *mitu*	*P*. *tuberosa*									
			0	0	0	1	2	2	2	2	2
			4	5	9	2	0	5	7	8	9
			0	0	1	8	7	3	6	8	5
Haplotype 1	66	0	**A**	**G**	**T**	**T**	**T**	**T**	**T**	**C**	**G**
Haplotype 2	6	11	**G**	**A**	**C**	**A**	**C**	**C**	**C**	**T**	**A**

### Genetic differentiation and diagnostic alleles

**Fig 4:**
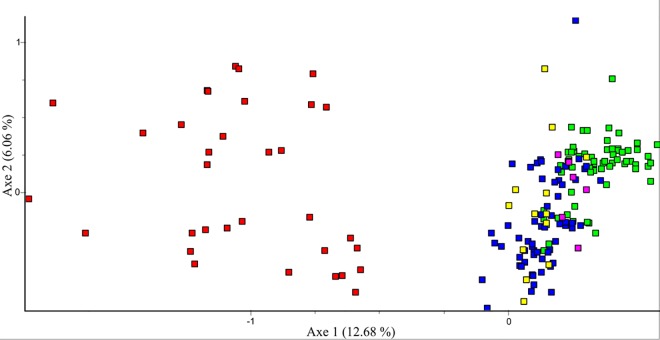
Factorial Correspondence Analysis using 14 microsatellite loci. General overview of the genetic relationships among Razor-billed Curassows (red); individuals assigned to Alagoas Curassow based on morphology, microsatellites, and mtDNA (green); individuals assigned to Alagoas Curassow based on morphology and microsatellites, but excluded by mtDNA analysis (purple); individuals pointed out as admixted by microsatellites, using Structure 98% threshold (yellow), and individuals assigned as hybrids based on morphology (blue).

Genetic differentiation between 33 Razor-billed Curassows and 66 animals identified as pure Alagoas Curassows (see results bellow) was high and extremely significant (*F*_ST_ = 0.281, P < 0.000). The two-dimensional overview resulted from the FCA based on multilocus microssatellites also indicated a clear divergence between the parental species, with the admixted individuals concentrated between the parental groups and around Alagoas individuals. This result was expected since backcrosses with Razor-billed Curassows have not occurred (Fig 4). The sample assigned to the Alagoas Curassow presented from 2 to 5 alleles per locus (3.0 ± 0.78), while the Razor-billed Curassow presented 2 to 14 alleles per locus (4.36 ± 3.27). Most of the alleles were present only in the Razor-billed Curassow, and seven alleles from five different loci were exclusive to the Alagoas Curassow. However, none of the loci was diagnostic of a species. The allele types found for each species are shown in S4 Table.

### Population status and sex identification

After our body of analyses and sex identification, our results can be summarized as follows: of the 148 individuals analyzed, 63 could be promptly identified as hybrids based on diagnostic morphological traits; of the 85 individuals presumably corresponding to Alagoas Curassow based on morphology alone, 72 (35 males and 37 females) were assigned to the Alagoas Curassow (using Structure with the chosen 98% threshold), and 13 (8 males and 5 females) revealed mixed ancestry based on the Bayesian modeling analyses. Finally, of the 72 animals that were attributed to the Alagoas Curassow by Structure, six (two males and four females) presented Razor-billed Curassow mtDNA. After removing those individuals, our analyses have resulted in 33 males and 33 females identified as pure Alagoas Curassows.

## Discussion

### Analyses power and hybrid detection

Analyses performed with our simulated data set indicated that Structure was more efficient to assign admixted individuals a hybrid origin, and using our markers, the performance of NewHybrids to attribute hybrids into classes was not satisfactory. As our main objective was to identify a group of Alagoas Curassow with minimal introgression probability, we have focused our discussion and management decisions on Structure results. With Structure, however, establishing a threshold level above which individuals can be considered pure is a critical step in hybridization analyses [5]. Most works use arbitrary limits from 90% to 98% [9, 26, 32, 44], but the appropriate value varies with each situation [5]. With lower thresholds (e.g., 90% or 95%), many loci are needed to detect hybridization in late backcross generations, leading most studies to focus only on the first backcross generation [26, 32, 44, 60]. Consequently, individuals presenting lower levels of admixture are assumed to have single-species ancestry, which makes it more likely to assign a hybrid a purebred origin [28]. Here we have used a high limit that permitted us to assign a hybrid origin even to second backcross generation with adequate efficiency, as demonstrated by the simulated data set. Despite the moderate number of loci, other factors may have contributed to the resolution of our analyses: first, the high and extremely significant population differentiation [28], and second, the certainty that the Razor-billed Curassows were purebred, which permitted us to use them as prior information, thereby increasing the accuracy of the Structure analyses.

However, using a high limit can also be problematic, and the risks of this decision must be evaluated. With high levels, it is not easy to distinguish between small amounts of admixture and natural polymorphisms shared by the target species, making it more likely to attribute a hybrid origin to a purebred animal [5, 28]. In the case of endangered species, a potential drawback of eliminating erroneously pure animals is increasing the loss of genetic variability, promoting even higher levels of genetic drift and inbreeding [5, 10]. Here, the risk of attributing a hybrid origin to a potential purebred relies especially on the 22 animals morphologically assigned to the Alagoas Curassow that were eliminated in the first stringent Structure analysis, as only nine of them were re-assigned a pure origin when the 98% threshold was established. However, as our simulated data set indicated a 3% probability of attributing a pure Alagoas Curassow to other classes using the 98% threshold, we considered the chances of error acceptable.

Although the average simulated *q* values obtained for each hybrid class were close to what would be theoretically expected (i.e., 0.5, 0.25, 0.125, and 0.06 for F1, first, second, and third backcrosses, respectively) [26], the highly overlapping ranges of values indicated that our markers could fail to correctly assign hybrids to their categories. This variation probably resulted from the number and characteristics of our microsatellites, for instance, the absence of diagnostic loci [5, 32]. We did not consider this variation to be a problem, as the variation found within hybrid classes did not interfere in our main purpose of identifying animals with pure or minimally mixed ancestry. Our data also revealed that among the admixed animals, an Alagoas phenotype is not indicative of lower admixture. Some of the animals assigned to the Alagoas group based on morphology presented *q* values that were similar to or even higher than some hybrids with Razor-billed Curassow phenotypical traits. This result suggests that admixed animals may inherit Razor-billed traits by chance, depending on the presence of certain alleles from the transcript loci.

The presence of Razor-billed mtDNA in some animals assigned to the Alagoas Curassow by the microsatellite analyses indicates a maternal ancestry of Razor-billed Curassow older than the power of our microsatellites and models to distinguish them as hybrids. Introgressed mtDNA molecules can persist in a population over many generations [10], and they revealed that the group of birds assigned to the Alagoas cluster by the Bayesian assignment procedure can include three categories: i) animals with low levels of mixed ancestry whose autosomic genomes have been “purified” over more than two generations of backcrosses but whose older hybrid origin can be identified due to the presence of Razor-billed mtDNA; ii) purified animals that by chance did not inherit Razor-billed mtDNA; and iii) a potentially pure lineage descending from the three founding Alagoas Curassows.

Although it is difficult to distinguish between individuals belonging to the last two categories, we are confident that we can safely identify a group without historical Razor-billed ancestry among the older animals. The Alagoas Curassow captive breeding program began in 1979, but hybridizations with Razor-billed Curassows occurred only from 1990 to 1999, a period in which all of the animals were still concentrated in the aviary located in Rio de Janeiro. Despite the absence of genealogical records, all birds in this breeding facility received a permanent metal band containing the name of the aviary, an individual number, and the year of birth. As they become sexually mature when they are three years of age, no F1 hybrids could have reproduced before 1993, which means that in the best scenario, backcrosses that occurred until 1999 (when this aviary was closed) could have reached the second generation, falling within a range of generations in which our markers could accurately detect hybridizations. Curassows are long-lived in captivity (20–25 years), and 23 animals born between 1990 and 1999 were still alive at the time of this study. In this group, all of the animals presenting the Alagoas phenotype (seven males and 10 females) were assigned to Alagoas cluster in the Bayesian analyses, and none of them had Razor-billed mtDNA. Thus, we believe they descend from the pure Alagoas pairs that were breeding at that time, constituting a group of animals that certainly preserve the original genome of the species; hereafter, we call these birds the “parental group”. This group also provided the best opportunity to identify the microsatellite alleles that certainly have Alagoas origin. Indeed, the alleles found in the “parental group” were the same found in the whole Alagoas cluster, excluding the animals with Razor-billed mtDNA.

### Conservation implications

The presence of a group of individuals with low levels of admixture, including an identifiable “parental group”, suggested that purebred Alagoas Curassows have survived through the 35 years of the captive breeding program, which reinforces the need for investments to save this species. This information permitted a reorganization of the breeding program to manage the three reproductive groups separately: the parental group; pure/purified animals not proved to belong to the parental group; and hybrids with or without Razor-billed phenotypical traits. It also gave support to the implementation of the first Brazilian official studbook for an endangered species, which will guarantee lineage control in future generations.

Of course, extreme efforts must be devoted to the reproduction of the parental group. Although these birds are approximately 16–20 years old, some of them are still reproducing, and during the 2012/2015 breeding season, at least 14 young descending from two males and three females of this group were born (young not included in this work). However, we must be aware that starting a new population exclusively from the parental group may imply another bottleneck and result in high risks of inbreeding depression. Thus, genetic variability, fertility and vulnerability to diseases must be carefully monitored in this lineage, and crosses with pure/purified individuals may be a prudent alternative, if necessary. The reproductive success of these animals in future generations will also determine the importance of the hybrids (see also [61]). If pure populations prove to be unviable, a certain amount of introgression might be the only solution to inbreeding depression, so that the hybrid population of Alagoas Curassow should be kept to form a genetic and demographic bank.

However, we consider it an important precaution to eliminate Razor-billed mtDNA from the breeding program. [10] have demonstrated that the introgression of mtDNA from Cattle was consistently correlated with phenotypic effects in free-roaming American Bison herds, as various genes in this molecule are transcribed. It causes smaller body sizes in some populations, a trait that is potentially associated with fitness, as the authors considered eliminating cattle mtDNA from Bison herds a wise management alternative for conserving the genomic integrity of the species [10]. Although the effects of Razor-billed mtDNA on the Alagoas Curassow phenotype remain an open question, considering this evidence, we suggest that at least the purified females that contained Razor-billed mtDNA should be prevented from reproducing, while the males should be used only if necessary.

In this new scenario, the Alagoas Curassow remains one of the most endangered birds in the world. As a study case, the purebred Alagoas Curassow population has a broader conservation relevance. To our knowledge, no species has ever survived such a severe bottleneck, followed by hybridization. Together with the Mauritius Kestrel, this situation provides a rare opportunity to study the recovery capability of a wild species that has been through bottlenecks of only two to three individuals and the effects on long-term population viability. Importantly, future projects envisioning the reintroduction of the Alagoas Curassow into the wild rely on the need for continuous protection of the few remaining fragments of the Atlantic Forest of northeastern Brazil, a region that concentrates a high number of endemisms as well as a dramatic number of vanishing and even recently extinct species [62].

## Supporting Information

S1 File Raw microsatellite data for individuals identified as Alagoas Curassow based on morphology. The data for 14 microsatellite loci are in three-digits Genepop file. Missing data are reported as 000000.
(TXT)

S2 File Raw microsatellite data for individuals identified as hybrids based on morphology. The data for 14 microsatellite loci are in three-digits Genepop file. Missing data are reported as 000000.
(TXT)

S3 File Raw microsatellite data for Razor-billed Curassow. The data for 14 microsatellite loci are in three-digits Genepop file. Missing data are reported as 000000.
(TXT)

S1 Table Assignment probabilities of individuals identified as Alagoas Curassow based on morphology to belong to Alagoas cluster in the preliminary stringent analyses. Values were estimated in Structure, and in NewHybrids with different prior combinations. Structure results represent the summarization of 20 runs with Clumpp. As individual classification did not change across three different runs of NewHybrids, we present only the results of the first run. Individuals indicated as potentially admixted using the 99% threshold are highlighted in yellow.
(XLSX)

S2 Table Proportional membership of 100 simulated genotypes of each parental and hybrid classes (F1, F2, F3, and first, second, third, and fourth generations of backcrosses with Alagoas Curassow), obtained with Structure based on the probability of belonging to two population clusters (K = 2), and with different prior combinations of NewHybrids. With Structure, individual admixture level was assessed based on the probability of belonging to Alagoas and Razor-billed Curassow, and results represent the summarization of 20 runs with Clumpp. With NewHybrids, values represent the probability of each individual to belong to pre-defined genotypic classes, and because individual classification did not change across three different runs, we present only the results of first run. Average, standard deviation, minimum, and maximum probability values are presented in the end of each column.
(XLSX)

S3 Table Admixture levels of 63 individuals identified as hybrids based on morphology obtained with Structure, and with NewHybrids using the best prior combination observed in the simulated dataset (Jeffreys X Jeffreys). Structure results represent the summarization of 20 runs with Clumpp. As individual classification did not change across three different runs of NewHybrids, we present only the results of the first run. Average, standard deviation, minimum, and maximum probability values are presented in the end of each column.
(XLSX)

S4 Table Alleles found for animals assigned to Alagoas Curassow, *Pauxi mitu* (Pm), and Razor-billed Curassow, *P*. *tuberosa* (Pt), across 14 microsatellite loci. Alleles are classified according to their sizes.
(DOCX)
