# Supplementary material for: Recovering the Genetic Identity of an Extinct-in-the-Wild Species: The Puzzling Case of the Alagoas Curassow
Source: PLoS One. 2017 Jan 5;12(1):e0169636. doi: 10.1371/journal.pone.0169636 (PMC5215914; doi:10.1371/journal.pone.0169636)
Supplement: S4 Table — Alleles are classified according to their sizes. (DOCX) [file pone.0169636.s007.docx]

| Locus | *Pauxi* 1-4 | | *Pauxi* 1-13 | | *Pauxi* 1-30 | | *Pauxi* 1-37 | | *Pauxi* 2-2 | | *Pauxi* 2-7 | | *Pauxi* 2-30 | | *Pauxi* 3-1 | | *Pauxi* 3-4 | | *Aburria* 21 | | *Aburria* 22 | | *Aburria* 36 | | *Aburria* 48 | | *Aburria* 49 | |
| --- | --- | --- | --- | --- | --- | --- | --- | --- | --- | --- | --- | --- | --- | --- | --- | --- | --- | --- | --- | --- | --- | --- | --- | --- | --- | --- | --- | --- |
| Species | *Pm* | *Pt* | *Pm* | *Pt* | *Pm* | *Pt* | *Pm* | *Pt* | *Pm* | *Pt* | *Pm* | *Pt* | *Pm* | *Pt* | *Pm* | *Pt* | *Pm* | *Pt* | *Pm* | *Pt* | *Pm* | *Pt* | *Pm* | *Pt* | *Pm* | *Pt* | *Pm* | *Pt* |
|  | 166 | 166 |  | 190 | 300 |  | 148 | 148 |  | 195 | 141 |  | 197 | 197 | 157 |  | 207 | 207 | 141 | 141 |  | 306 |  | 448 | 87 | 87 | 181 | 181 |
|  |  | 170 |  | 194 | 302 | 302 | 180 | 180 |  | 225 |  | 147 | 201 | 201 |  | 161 | 217 | 217 | 145 | 145 | 308 | 308 | 452 | 452 | 89 | 89 | 205 | 205 |
|  | 174 | 174 |  | 202 | 306 | 306 |  |  | 227 | 227 | 149 | 149 | 205 |  | 165 | 165 | 219 | 219 | 153 |  | 310 |  | 454 | 454 |  |  | 225 | 225 |
|  |  | 178 |  | 206 |  | 310 |  |  | 233 | 233 |  | 151 |  |  |  | 169 |  |  |  |  |  | 312 |  |  |  |  |  |  |
|  |  | 182 | 210 | 210 |  |  |  |  | 237 | 237 | 153 | 153 |  |  | 171 | 171 |  |  |  |  |  | 314 |  |  |  |  |  |  |
|  |  | 186 |  | 214 |  |  |  |  |  | 241 |  |  |  |  | 173 |  |  |  |  |  | 326 | 326 |  |  |  |  |  |  |
|  | 190 | 190 |  | 222 |  |  |  |  |  |  |  |  |  |  |  |  |  |  |  |  |  |  |  |  |  |  |  |  |
|  |  | 206 |  | 226 |  |  |  |  |  |  |  |  |  |  |  |  |  |  |  |  |  |  |  |  |  |  |  |  |
|  |  |  |  | 230 |  |  |  |  |  |  |  |  |  |  |  |  |  |  |  |  |  |  |  |  |  |  |  |  |
|  |  |  |  | 234 |  |  |  |  |  |  |  |  |  |  |  |  |  |  |  |  |  |  |  |  |  |  |  |  |
|  |  |  |  | 238 |  |  |  |  |  |  |  |  |  |  |  |  |  |  |  |  |  |  |  |  |  |  |  |  |
|  |  |  |  | 246 |  |  |  |  |  |  |  |  |  |  |  |  |  |  |  |  |  |  |  |  |  |  |  |  |
|  |  |  | 250 | 250 |  |  |  |  |  |  |  |  |  |  |  |  |  |  |  |  |  |  |  |  |  |  |  |  |
|  |  |  |  | 252 |  |  |  |  |  |  |  |  |  |  |  |  |  |  |  |  |  |  |  |  |  |  |  |  |
|  |  |  | 254 |  |  |  |  |  |  |  |  |  |  |  |  |  |  |  |  |  |  |  |  |  |  |  |  |  |
|  |  |  | 262 |  |  |  |  |  |  |  |  |  |  |  |  |  |  |  |  |  |  |  |  |  |  |  |  |  |
|  |  |  | 266 |  |  |  |  |  |  |  |  |  |  |  |  |  |  |  |  |  |  |  |  |  |  |  |  |  |
|  |  |  |  |  |  |  |  |  |  |  |  |  |  |  |  |  |  |  |  |  |  |  |  |  |  |  |  |  |
| Total | 3 | 8 | 5 | 14 | 3 | 3 | 2 | 2 | 3 | 6 | 3 | 4 | 3 | 2 | 4 | 4 | 3 | 3 | 3 | 2 | 3 | 5 | 2 | 3 | 2 | 2 | 3 | 3 |
